# Postnatal virtual and home visits by midwives during COVID-19 pandemic

**DOI:** 10.18332/ejm/120971

**Published:** 2020-04-28

**Authors:** Maria Gjoni, Efstathia M. Alevizou

**Affiliations:** 1 Midwifery Department, University of West Attica, Athens, Greece

**Keywords:** midwives, COVID-19, virtual visits, postnatal, home visits

**Dear Editor,**

During the COVID-19 pandemic postnatal midwifery-led care remains a key public health intervention for women and their families^1,2^. Home or virtual visits by community midwives should be provided to reduce the number of times women and newborns need to leave their home^1^. In order to comply with social distancing requirements, community midwives could deliver home or virtual visits and individualized postnatal care, according to the needs of the mother and the newborn^1^. Midwifery-led postnatal home visiting could raise consciousness in parents on establishing breastfeeding, family planning and contraception after birth, preparation of milk formula, smoke-free environments at home, home hygiene, and care of the neonatal – especially after NICU discharge^3^. Midwifery-led virtual visits via videoconferencing decrease the number of visits to health facilities and enable mothers to be consulted immediately and from their own home^4^. Virtual visits have been demonstrated to be as safe as in-person visits^5^. Therefore, as early as possible, community midwives (especially those serving rural and remote areas) should receive all the relevant technology equipment and training regarding remote consultation^5^.

The World Health Organization (WHO) recommends at least four postnatal visits for all mothers and newborns, on day 1 (first 24 hours), on day 3 (48–72 hours), between days 7–14 and six weeks after birth^6^. However, a face-to-face consultation is required for physical examination and/or screening of mother and the newborn^1^. Therefore, home visiting should be prioritized for women with psychosocial vulnerabilities, operative birth, premature or low birthweight baby and other medical or neonatal complications^1^. Awareness should be raised about exposure to COVID-19 during a home visit, where midwives should abide by strict infection control equipment and procedures when entering and leaving homes^1^.

Midwifery-led services are vital during the postpartum period for women suspected or confirmed with COVID-19. Parenting and breastfeeding support can be offered through face-to-face or virtual visits^1^. Μothers with COVID-19 should be advised to establish breastfeeding or to express breastmilk, applying appropriate infection prevention and control measures^2,7^. All breastfeeding women should be shown how to hand-express their breast milk and be advised on how to correctly store and freeze it. Breastfeeding counseling, neonatal care, family planning counseling, psychosocial parent support, practical feeding support and home hygiene should be provided to all mothers and their families, whether they or their children have suspected or confirmed COVID-19^7^.

Community midwives are a valuable resource either providing face-to-face visits or virtual visits^8^. They need adequate support^8^ to provide quality, holistic, women-centered postnatal care to mothers and newborns^9^ in these exceptionally complex circumstances in order to promote safe and respectful^10^ family-centered care, during periods of crisis including the current COVID-19 pandemic.

## References

[1] Royal College of Obstetricians and Gynaecologists, Royal College of Midwives Guidance for antenatal and postnatal services in the evolving coronavirus (COVID-19) pandemic.

[2] International Confederation of Midwives Women’s Rights in Childbirth Must be Upheld During the Coronavirus Pandemic.

[3] Mokhtari F, Bahadoran P, Baghersad Z (2018). Effectiveness of postpartum homecare program as a new method on mothers' knowledge about the health of the mother and the infant. Iranian J Nursing Midwifery Res.

[4] Seguranyes G, Costa D, Fuentelsaz-Gallego C (2014). Efficacy of a videoconferencing intervention compared with standard postnatal care at primary care health centres in Catalonia. Midwifery.

[5] Pflugeisen BM, McCarren C, Poore S, Carlile M, Schroeder R (2016). Virtual Visits: Managing prenatal care with modern technology. MCN Am J Matern Child Nurs.

[6] World Health Organization WHO recommendations on postnatal care of the mother and newborn.

[7] World Health Organization Clinical management of severe acute respiratory infection (SARI) when COVID-19 disease is suspected.

[8] Vermeulen J, Jokinen M (2020). The European Midwives Association call for action to protect our midwives in delivering best care amidst the COVID-19 pandemic. Eur J Midwifery.

[9] Smith HA (2020). Impact of COVID-19 on neonatal health: Are we causing more harm than good?. Eur J Midwifery.

[10] Vivilaki VG, Asimaki E (2020). Respectful midwifery care during the COVID-19 pandemic. Eur J Midwifery.

